# Transanal Intrusion Avoided: A Rare Case of Pediatric Accidental Penetrating Gluteal Trauma Induced by a Pencil Without Internal Perforation

**DOI:** 10.7759/cureus.88811

**Published:** 2025-07-26

**Authors:** Sania Shahid, Bachir Farzat, Usman Mansoor, Munir Ahmad, Razan Ismail, Fathimathul Henna, Aqsha Shahid, Eman Ahmad

**Affiliations:** 1 Pediatric Emergency Medicine, Al Jalila Children's Specialty Hospital, Dubai, ARE; 2 Pediatric Surgery, Al Jalila Children's Specialty Hospital, Dubai, ARE; 3 Medicine, Dubai Medical University, Dubai, ARE; 4 Medicine, The University of Georgia, Tbilisi, GEO; 5 Medicine, Fatima Memorial Hospital College of Medicine and Dentistry, Lahore, PAK

**Keywords:** diagnostic laparoscopy, emergency, endoscopy, laparoscopy, pediatric endoscopy, pencil, pencil injury, surgery, trauma, trauma pediatric

## Abstract

Penetrating injuries to the gluteal region are rare in children and often pose a diagnostic and therapeutic challenge due to the proximity of vital pelvic structures such as the rectum, bladder, and major vessels. Injuries caused by common classroom supplies like pencils are uncommon, but their ability to penetrate deeply into the pelvis warrants high suspicion for visceral injury. Here, we report a case of an 11-year-old previously healthy boy who suffered a left-sided gluteal injury after unintentionally sitting on a pencil placed upright on a chair as a prank during school hours. Initial assessment showed a puncture wound without external bleeding or signs of peritonitis. Contrast-enhanced computed tomography (CT) of the pelvis revealed a linear foreign body measuring 12.4 cm in length passing through the ischiorectal fossa and appearing to penetrate the posterior rectosigmoid wall, with the tip located inside the lumen. Despite imaging suggesting rectal perforation, diagnostic laparoscopy and colonoscopy showed no mucosal injury, contamination, or any signs of perforation. The foreign body was successfully removed through the entry wound, and the patient was managed conservatively with intravenous antibiotics and close clinical monitoring. Postoperatively, he developed abdominal pain and elevated inflammatory markers, with ultrasound revealing a small retrorectal fluid collection that improved with ongoing conservative care. He fully recovered and remained asymptomatic on follow-up. The aim of the report is to highlight a rare case and underscore the importance of correlating imaging findings with surgical and endoscopic assessments in pediatric trauma and support a purposeful, minimally invasive targeted treatment approach in the absence of obvious perforation or systemic compromise.

## Introduction

Penetrating injuries to the gluteal region are uncommon in the pediatric population and represent a special subset of trauma cases. The intricate anatomy of the gluteal and pelvic areas, including the proximity to the rectum, bladder, pelvic vasculature, sciatic nerve, and pelvic bones, makes assessment and management challenging [1]. Although gluteal trauma is more often reported in adults due to physical assault or occupational injuries, its presentation in children is unique and frequently overlooked in the literature [1].

In pediatric patients, penetrating injuries are primarily caused by falls onto sharp objects, unintentional impalement, or, less frequently, abuse. Impalement by common stationary items, such as pencils, is uncommon, but these objects can cause deep, trajectory wounds that may involve vital structures. Complicating the situation is the often-misleading external appearance; small entry wounds can mask the extensive internal injury [2].

Radiological imaging, especially computed tomography (CT), plays a vital role in tracing the trajectory and potential visceral involvement. However, CT can sometimes misjudge the severity or depth of penetration, especially when it comes to differentiating the mucosal indentation from true transmural perforation [3]. When the rectosigmoid area is affected, there is high concern for contamination and the need for bowel diversion, but this must be weighed against the morbidity linked to unnecessary surgery in children [3].

In this case report, we present a rare case of an 11-year-old boy who suffered a gluteal impalement injury from a pencil. CT findings demonstrated penetration into the rectosigmoid colon, but the diagnostic laparoscopy and colonoscopy revealed no perforation. The patient was managed conservatively without the need for colostomy or laparotomy. This case highlights the importance of correlating imaging with intraoperative and endoscopic findings, supporting a conservative approach in selected pediatric patients.

## Case presentation

An 11-year-old previously healthy boy arrived at the emergency department after a penetrating injury to the left buttock at his school. According to the patient and school staff, a classmate had placed a pencil upright on a chair as a prank, and the patient unknowingly sat on it, resulting in a penetrating wound to the left buttock.

Upon arrival at the emergency department, the patient was alert and hemodynamically stable. Vital signs showed a blood pressure of 115/78 mmHg, a pulse of 82 bpm, a temperature of 37°C, a respiratory rate of 24 breaths/minute, and an SpO₂ of 98%, all within normal limits. Physical examination revealed a puncture wound in the inferomedial aspect of the left gluteal region with no active bleeding or neurological or vascular compromise. The abdominal exam showed a soft, non-distended abdomen with normal bowel sounds. The digital rectal exam was delayed due to concerns for rectal involvement.

Laboratory tests at presentation showed a normal hemoglobin level of 12.7 g/dL (reference: 11.5-15.5 g/dL), a white blood cell count of 9.8×10⁹/L (reference: 5.0-13.0 ×10⁹/L), and a creatinine level of 0.47 mg/dL (reference: 0.52-0.69 mg/dL) (Table 1).

**Table 1:** Serial laboratory values during hospitalization WBC: white blood cell; CRP: C-reactive protein; NR: not reported

Parameter	On Admission	Post-op Day 2	Discharge	Reference Range
WBC (×10³/μL)	9.8	17.9	8.4	5.0–13.0
Hemoglobin (g/dL)	12.7	12.5	12.4	11.5–15.5
CRP (mg/L)	2	211	8	<5
Procalcitonin (ng/mL)	0.04	0.6	NR	<0.5
Creatinine (mg/dL)	0.47	0.47	0.50	0.52–0.69
Sodium (mmol/L)	139	140	NR	136–145
Potassium (mmol/L)	4.1	4.2	NR	3.5–5.1

**Figure 1:**
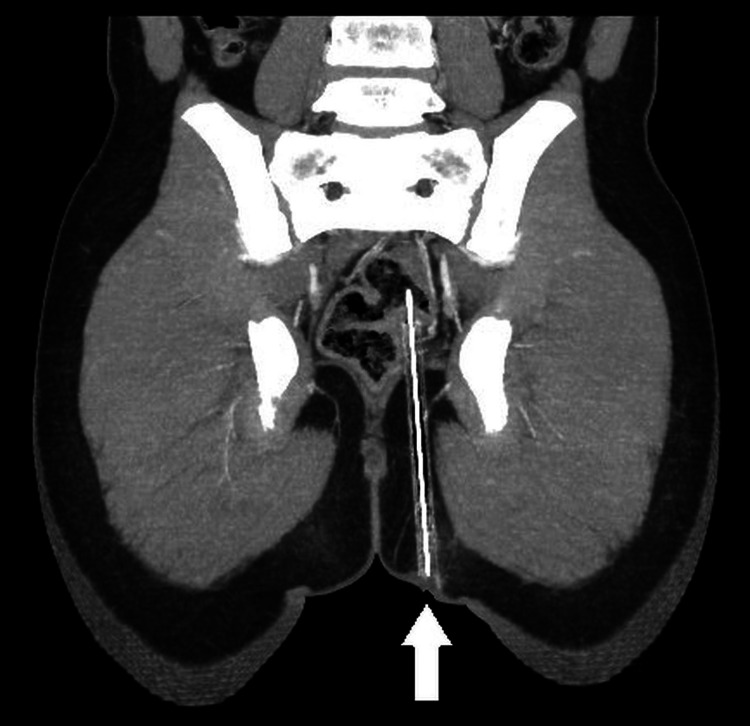
Coronal CT scan shows a hyperdense linear foreign body (white arrow) extending from the inferomedial left gluteal region into the pelvis. The tip is seen approaching the posterior wall of the rectosigmoid junction, with no signs of free air or fluid.

**Figure 2:**
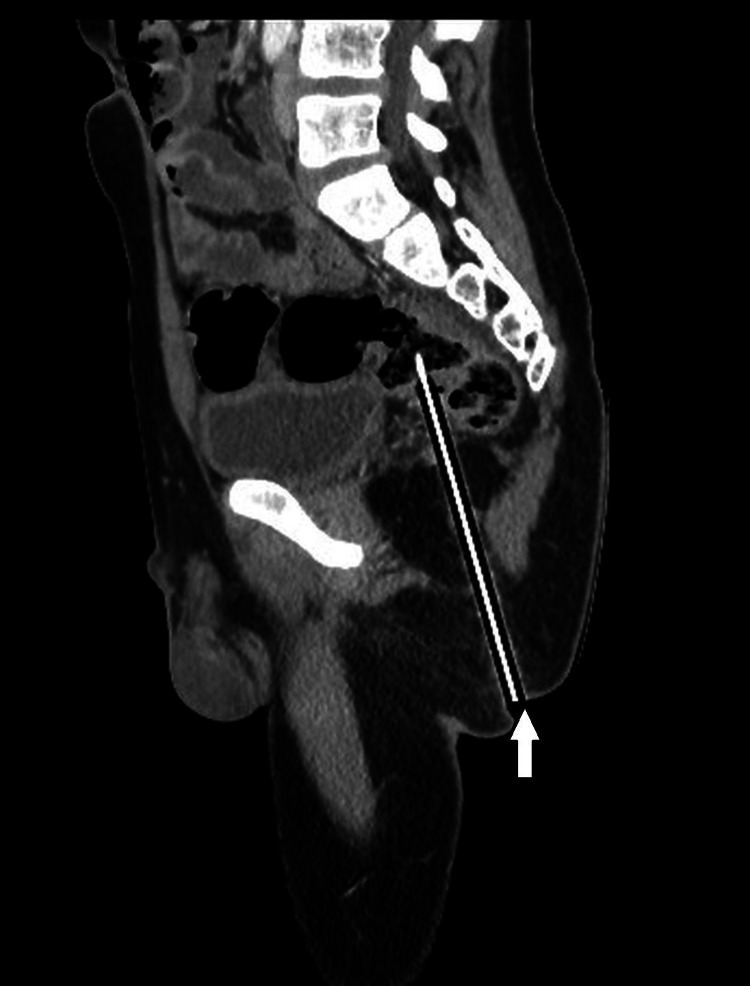
Sagittal CT reconstruction shows the full trajectory of the pencil (white arrow) with the tip appearing to project toward or into the rectosigmoid lumen, indicating a possible intraluminal position.

**Figure 3:**
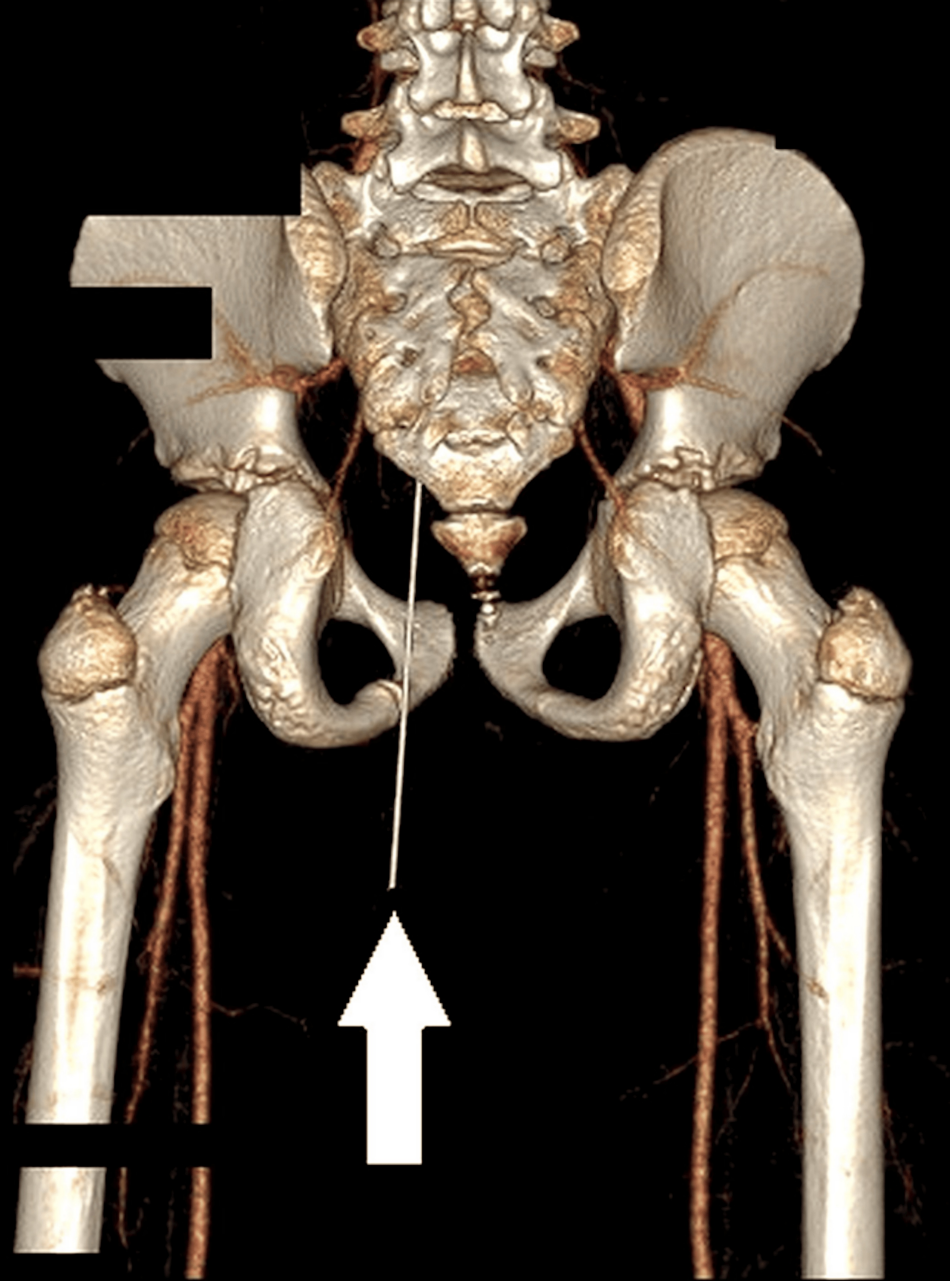
Three-dimensional volume-rendered CT reconstruction illustrating the spatial course of the foreign body (white arrow) from the left buttock through the pelvis, clearly visualized in relation to surrounding bony structures and soft tissue anatomy.

CT pelvis with contrast revealed an elongated hyperdense foreign object measuring 12.4 cm × 6.7 mm traversing the ischiorectal and ischioanal fossae, with the tip seeming to penetrate the posterior rectosigmoid bowel wall and lie intraluminally. There was no evidence of free fluid, air in the peritoneal cavity (pneumoperitoneum), or vascular injury. Coronal CT imaging showed an elongated foreign object penetrating from the gluteal region toward the posterior rectosigmoid wall (Figure 1). The sagittal CT view demonstrated the tip to be intraluminal in the rectosigmoid colon (Figure 2). In addition to standard CT scans, a 3D volume-rendered CT reconstruction was performed, which provided a clearer view of the course and depth of the foreign body in relation to the bony pelvis and soft tissue structures (Figure 3).

A multidisciplinary team involving pediatric surgery and gastroenterology recommended diagnostic laparoscopy and colonoscopy. The patient's father provided informed consent. Under general anesthesia, laparoscopy showed no signs of perforation, contamination of the peritoneum, or bowel contents spillage. Colonoscopy revealed intact mucosa across the rectosigmoid junction. The pencil was then carefully removed through the gluteal entry wound. No active bleeding was observed. The wound was cleaned and dressed in a regular gauze, and the abdomen was reinspected before port closure.

**Figure 4:**
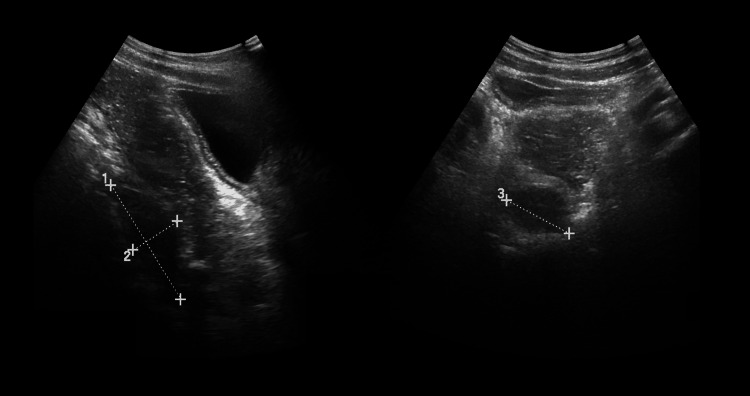
Ultrasound image of the pelvis showing a retrorectal fluid collection measuring approximately 6.5 × 2.5 × 3.4 cm (30.45 mL) with internal debris. This collection was managed conservatively with intravenous antibiotics and follow-up imaging.

Postoperatively, the patient was kept on nil per oral (NPO) and started on intravenous piperacillin-tazobactam and metronidazole. On day two postoperatively, he began experiencing abdominal discomfort and mild hypotension (BP 90/50 mmHg). Inflammatory markers appeared elevated: CRP 211 mg/L (reference: <5 mg/L) and WBC count of 17.9×10⁹/L (reference: 5.0-13.0 ×10⁹/L). Abdominal ultrasound showed a retrorectal collection measuring approximately 16-20 mL with internal debris. Follow-up imaging later revealed the collection increased to 30.45 mL (6.5 × 2.5 × 3.46 cm) (Figure 4). As the child remained stable after adequate hydration, he was managed conservatively. The surgical team decided to observe the CRP trend, as procalcitonin was mildly elevated at 0.6 ng/mL (reference <0.5 ng/mL), and the child was responding well to antibiotics.

By day five postoperatively, the patient's abdominal symptoms had subsided. Oral intake was resumed, and the patient tolerated it well. A repeat ultrasound showed a reduction of the collection to 2.7 × 2.6 × 1.4 cm. He was discharged on oral antibiotics and paracetamol. During follow-up after one week of discharge, he was asymptomatic, and an ultrasound revealed complete resolution of the retrorectal collection. Complete wound healing was observed, with no signs of infection.

## Discussion

Penetrating gluteal injuries in children are rare, but they can involve deep pelvic structures due to the complex anatomy and soft tissue elasticity. The path of impalement injuries can be misleading outward, making radiological imaging necessary for evaluation [4]. In our case, CT imaging revealed rectosigmoid perforation caused by the foreign object, raising concerns about fecal contamination and necessitating rerouting.

CT is frequently used in trauma imaging; however, its accuracy in evaluating the integrity of the intestinal wall, particularly in small pediatric anatomy, may be limited. In particular, distinguishing between mucosal indentation and full-thickness perforation is often not possible without direct visualization [3]. This uncertainty in diagnosis warrants careful correlation with operative findings. In this case, despite CT indicating rectosigmoid perforation, direct visualization during laparoscopy and colonoscopy revealed intact mucosa with no signs of fecal contamination or injury, guiding the team away from invasive treatment.

Historically, any concern of rectal injury requires laparotomy with colostomy due to the risk of pelvic sepsis [5]. However, many pediatric case reports and small series have demonstrated that, in stable patients without fecal contamination or peritonitis, conservative management can be a safe and effective option [5].

In contrast to this study, Mercer et al. (1992) observed a 32% incidence of visceral or vascular injury in upper-zone gluteal penetrations, often requiring surgical exploration [6].

The retrorectal fluid collection seen postoperatively was managed conservatively with antibiotics. A plausible explanation for this collection could be a reactionary inflammatory exudate caused due to tissue trauma from the penetrating pencil injury. Such trauma can lead to capillary leakage, fibrin deposition, and sterile fluid collection, resulting in localized inflammation. This aligns with previous experiences, indicating that small, localized presacral collections without systemic instability do not require drainage [7].

In our case, the patient's CRP level rose significantly from 2 mg/L preoperatively to 211 mg/L postoperatively, then gradually decreased to 8 mg/L. Procalcitonin levels similarly increased from 0.04 ng/mL to 0.6 ng/mL, without any clinical signs of sepsis, such as fever, hemodynamic instability, or leukocytosis. These trends are consistent with postoperative sterile inflammatory responses rather than infection. Previous studies in pediatric surgical patients have demonstrated that CRP and procalcitonin levels can rise markedly following surgery due to tissue injury and inflammatory activation, even in the absence of infection [8,9]. This aligns with our clinical decision to manage conservatively, as the child remained stable and the retrorectal collection showed no features of abscess or systemic infection. The eventual normalization of inflammatory markers and resolution of the collection further supported a non-septic postoperative course.

This case highlights how relying solely on imaging may lead to overtreatment. Instead, combining radiological data with real-time surgical assessment allowed for an individualized, minimally invasive approach. The successful outcome, without complications or rerouting, further demonstrates the safety and effectiveness of personalized decision-making in stable pediatric patients.

## Conclusions

This case highlights the complexities of diagnosing and managing penetrating gluteal trauma in pediatric patients, especially when imaging suggests rectal involvement. In our patient, CT initially raised concerns for a rectosigmoid perforation; however, minimally invasive assessment with laparoscopy and colonoscopy revealed no evidence of visceral injury. The decision to pursue conservative, non-diversional management, guided by endoscopic and intraoperative findings, resulted in full clinical recovery without the need for unnecessary surgical intervention.

Physicians should exercise caution when solely interpreting imaging results and consider a personalized, multidisciplinary approach that balances the need for intervention with the risk of overtreatment. In stable pediatric patients without signs of perforation or sepsis, conservative management with close clinical monitoring may be both safe and effective. By integrating clinical judgment with operative findings, we were able to safely manage the injury and avoid an unnecessary laparotomy.
